# Bioactive Hydrogels Based on Tyramine and Maleimide Functionalized Dextran for Tissue Engineering Applications

**DOI:** 10.3390/gels10090566

**Published:** 2024-08-30

**Authors:** Lin Zhong, Alma Tamunonengiofori Banigo, Bram Zoetebier, Marcel Karperien

**Affiliations:** Department of Developmental BioEngineering, Faculty of Science and Technology, TechMed Centre, University of Twente, Drienerlolaan 5, 7522 NB Enschede, The Netherlands; lin.zhong@maastrichtuniversity.nl (L.Z.); a.tamunonengioforibanigo@utwente.nl (A.T.B.); b.zoetebier@utwente.nl (B.Z.)

**Keywords:** hydrogel, dextran, RGD peptide, adipogenic differentiation

## Abstract

Hydrogels are widely used in tissue engineering due to their ability to form three-dimensional (3D) structures that support cellular functions and mimic the extracellular matrix (ECM). Despite their advantages, dextran-based hydrogels lack intrinsic biological activity, limiting their use in this field. Here, we present a strategy for developing bioactive hydrogels through sequential thiol–maleimide bio-functionalization and enzyme-catalyzed crosslinking. The hydrogel network is formed through the reaction of tyramine moieties in the presence of horseradish peroxidase (HRP) and hydrogen peroxide (H_2_O_2_), allowing for tunable gelation time and stiffness by adjusting H_2_O_2_ concentrations. Maleimide groups on the hydrogel backbone enable the coupling of thiol-containing bioactive molecules, such as arginylglycylaspartic acid (RGD) peptides, to enhance biological activity. We examined the effects of hydrogel stiffness and RGD concentration on human mesenchymal stem cells (hMSCs) during differentiation and found that hMSCs encapsulated within these hydrogels exhibited over 88% cell viability on day 1 across all conditions, with a slight reduction to 60–81% by day 14. Furthermore, the hydrogels facilitated adipogenic differentiation, as evidenced by positive Oil Red O staining. These findings demonstrate that DexTA–Mal hydrogels create a biocompatible environment that is conducive to cell viability and differentiation, offering a versatile platform for future tissue engineering applications.

## 1. Introduction

Dextran (Dex) is a polysaccharide derived from natural glucose sources through bacterial synthesis, comprising α-(1,6) linked D-glucose units [1]. This biocompatible, hydrophilic, non-toxic, and biodegradable polymer is extensively used in biomedical applications [2,3,4,5,6]. Dextran can serve as a vehicle for drug delivery [7,8] and as a scaffold [9] for soft tissue engineering, particularly in the form of hydrogels functionalized with bioactive molecules [10,11,12]. Dextran has three active hydroxyl groups on each D-glucose unit that can be chemically modified to add various functional groups, allowing for the creation of dextran with specific properties based on the modifications. These chemical modifications are often used to form hydrogels through different crosslinking methods (e.g., homo-, hetero-, enzymatic, and photoreactive crosslinking), or to functionalize scaffolds with bioactive molecules [10,11,12].

Currently, there is growing interest in developing hydrogels with enzymatic crosslinking (EC) due to the mild nature of this type of reaction, which allows for injectable in situ gelating hydrogels [13]. The EC mechanism used in this study works under physiological conditions, utilizing horseradish peroxidase (HRP) as a catalyst and hydrogen peroxide (H_2_O_2_) as the substrate. The crosslinking rate can be fine-tuned from seconds to minutes, with no need for additional materials, including organic solvents [14,15]. Previously, Jin et al. produced in situ forming dextran hydrogels using the dextran–tyramine (DexTA) conjugates, which demonstrated favourable characteristics for both controlled drug delivery and injectable hydrogel applications [16,17,18]. However, these hydrogels exhibited limited biological activity due to the absence of bioactive sites for cell interaction, thus limiting their ability to biologically mimic the extracellular matrix (ECM).

This limitation can be addressed by incorporating biopolymers such as heparin and/or hyaluronic acid [19,20], or by attaching functional groups to immobilize bioactive molecules. These bioactive molecules may include cell adhesion ligands, growth factors, and signalling peptides, which can enhance cell adhesion, function, and behaviour [21].

Maleimides are commonly explored for cysteine (Cys) conjugation, particularly in the production of antibody–drug conjugates via the Michael-type addition reaction [22]. Maleimide-functionalized polysaccharides can be crosslinked through the thiol–maleimide reaction to develop hydrogels for tissue scaffold biofabrication and drug delivery [23,24,25]. Additionally, this thiol-reactive group is often used to ligate peptides and proteins to scaffold materials [26,27], thereby controlling a wide range of cellular behaviours [21]. Recently, dextran derivatives bearing maleimide moieties have been utilized for the fabrication and functionalization of hydrogels, which can be further modified with RGD peptides to influence cellular behaviour through cell–biomaterial interactions [28,29]. Moreover, polymers such as polyethylene glycol (PEG)–maleimide and hyaluronic acid–maleimide have been explored for producing biocompatible hydrogels based on the thiol–maleimide reaction [30,31]. However, the rapid gelation kinetics of these specific thiol-reactive polymers often lead to inadequate mixing and heterogeneous gelation, as the polymer network forms before thorough manual mixing of the components is possible [30].

To address this challenge, we developed an efficient method for synthesizing a bifunctional dextran, named dextran–tyramine–maleimide (DexTA–Mal), which is used to create a bioactive hydrogel through sequential thiol–maleimide biofunctionalization and enzyme-catalysed crosslinking. The tyramine moieties enable enzymatic crosslinking to form stable hydrogel networks, while the maleimide groups allow for the coupling of thiol-containing biomolecules to the polymer backbone. We studied the gelation time of the hydrogel and its physical properties, such as the storage modulus and swelling ratios. Furthermore, we demonstrated the hydrogel’s bioactivation capacity by conjugating two model biomolecules, a cysteine-containing peptide and BSA, to the DexTA–Mal hydrogel. Finally, we prepared a bioactive hydrogel by incorporating a CRGDS peptide known for promoting cell adhesion, and characterized its impact on the viability and differentiation of human mesenchymal stem cells (hMSCs) under varying stiffness conditions.

## 2. Results and Discussion

### 2.1. Synthesis of DexTA–Mal

**Figure 1:**
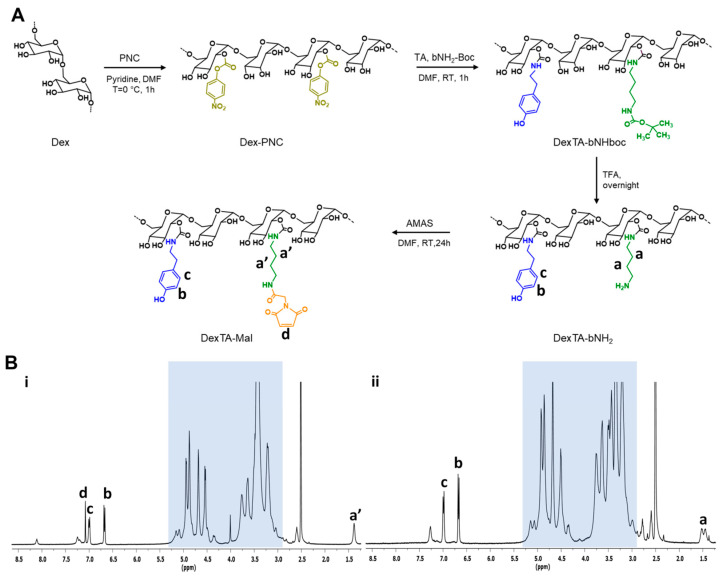
Synthesis and characterization of DexTA–Mal. (**A**) Schematic of synthesis route for DexTA–Mal, (**B**) ^1^H-NMR spectra of DexTA–Mal (**i**), and DexTA–bNH_2_ (**ii**) in DMSO-d_6_, the dextran region is shaded and the letters corresponding to the protons are marked in the chemical structures above. TFA, trifluoroacetic acid; bNH_2_, butylamine; boc, tert-butyloxycarbonyl; AMAS, n-(α-maleimidoacetoxy)succinimide ester [32].

The synthesis of DexTA–Mal was carried out in four distinct steps (Figure 1A). Initially, the hydroxyl groups of dextran were activated using PNC to form carbonate intermediates, which reacted with tyramine and n-boc-1,4-diaminobutane. The Boc protecting group was subsequently removed using trifluoroacetic acid to yield DexTA–bNH_2_. The primary amine group of DexTA–bNH_2_ served as a reactive site for conjugation with the NHS ester of a maleimide derivative. DexTA–Mal was then synthesized by reacting DexTA–bNH_2_ with n-(α-maleimidoacetoxy) succinimide ester (AMAS) in N,N-Dimethylformamide (DMF).

Successful conjugation was confirmed by proton nuclear magnetic resonance (^1^H-NMR), which identified the central methylene protons of butylamine at 1.40–1.60 ppm and peaks from the maleimide moiety at 7.08 ppm (Figure 1B). The degree of substitution (DS) refers to the average number of functional groups substituted per repeating structural unit (such as a glucose unit in polysaccharides like dextran) within a polymer. In the context of dextran–tyramine–maleimide (DexTA–Mal) hydrogels, the DS value would indicate the average number of tyramine or maleimide groups attached to the dextran backbone. The DS value is crucial because it influences the polymer’s chemical properties, such as its reactivity, crosslinking density, and overall functionality in applications like hydrogel formation. The DS was calculated, yielding a DS value of 5.2 for the free amine group and 5.0 for the maleimide group, indicating that approximately 100% of the primary amine groups in DexTA–bNH_2_ were successfully modified with maleimide. The DS values for tyramine (9.0) and maleimide (5.0) were also determined with ^1^H-NMR.

### 2.2. Hydrogel (Bio-)Functionalization

**Figure 2:**
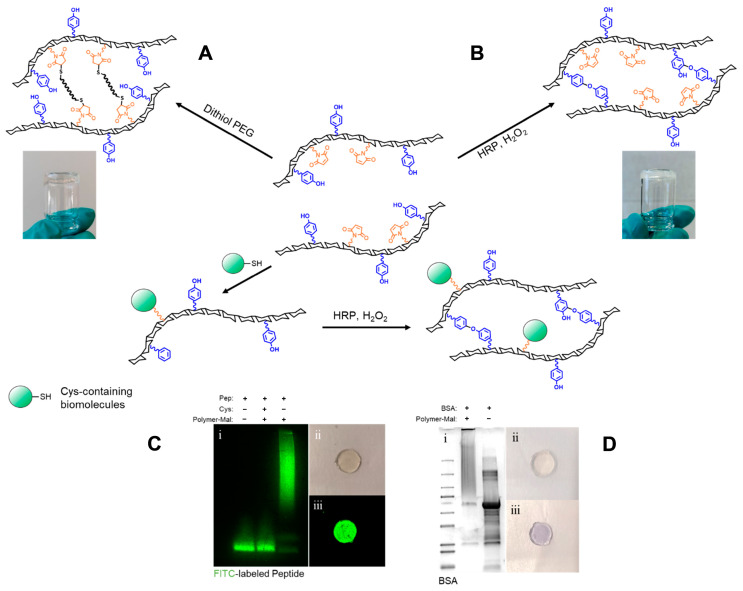
Schematic representation of the DexTA–Mal polymers, capable of forming a hydrogel (**A**) with PEG dithiol (**B**) or via enzymatic crosslinking of the tyramine moieties. Biofunctionalization is demonstrated with (**C**) a fluorescent peptide (**i**) SDS-PAGE gel analysis and fluorescence visualization of FITC-labeled peptide after conjugating to DexTA-Mal (**ii**) bright-field and (**iii**) fluorescence imaging of FITC-labeled peptide in hydrogel (**D**) and with BSA (**i**) Coomassie blue-stained SDS-PAGE gel analysis of BSA after conjugating to DexTA-Mal, (**ii**) digital photograph of BSA-loaded hydrogel before and (**iii**) after coomassie blue staining. (Pep: FITC-labeled peptide; Cys: cysteine; Polymer-Mal: DexTA-Mal) [32].

Biomolecule-functionalized hydrogels represent a powerful tool for guiding cell behaviour and fates within three dimensional environments, making them highly valuable in tissue engineering applications [33]. A common strategy for decorating hydrogels with biomolecules such as peptides and proteins involves covalent attachment to the hydrogel matrix, thereby facilitating their bioactive presentation to cells. Moreover, the thiol-reactive groups on maleimide-functionalized polymers are useful not only for attaching biomolecules to the scaffold, but also for crosslinking through thiol–maleimide reactions to form hydrogels (Figure 2A). However, the rapid gelation kinetics of maleimide-functionalized polymers often result in poor mixing and uneven gelation [30]. In this study, we synthesized a bifunctional dextran, DexTA–Mal, incorporating a tyramine moiety to enable the formation of a homogeneous hydrogel (Figure 2B), while the maleimide groups were used to attach thiol-containing biomolecules. To assess the attachment of biomolecules to the polymer, we used a cysteine-containing FITC-labelled peptide and bovine serum albumin (BSA), the latter of which contains a single free cysteine residue (Cys34) out of 583 amino acids. Model biomolecule conjugation was achieved by reacting a 10-molar equivalent of DexTA–Mal with the peptide and BSA at 4 °C overnight in PBS. SDS-PAGE analysis confirmed the successful conjugation, as both peptide–dextran and BSA–dextran conjugates displayed a smeared band with an increased molecular weight relative to the unconjugated peptide and BSA (Figure 2). This observation suggests that the thiol–maleimide chemistry employed for immobilization was efficient under these mild reaction conditions.

These biomolecule-functionalized polymers were further utilized to create stable hydrogels through tyramine-mediated crosslinking. The tethering of the peptide to the hydrogel matrix was confirmed using a fluorescent imaging system, while the presence of BSA within the hydrogel was visualized via Coomassie brilliant blue staining (Figure 2). The ability of DexTA–Mal to form hydrogels while effectively coupling with various Cys-containing biomolecules highlights its potential as a versatile platform for fabricating bioactive hydrogels. This capability was demonstrated in our study by tethering CRGDS to the hydrogels, thereby directing cell fates and behaviour.

### 2.3. Hydrogel Formation and Gelation Time

**Figure 3:**
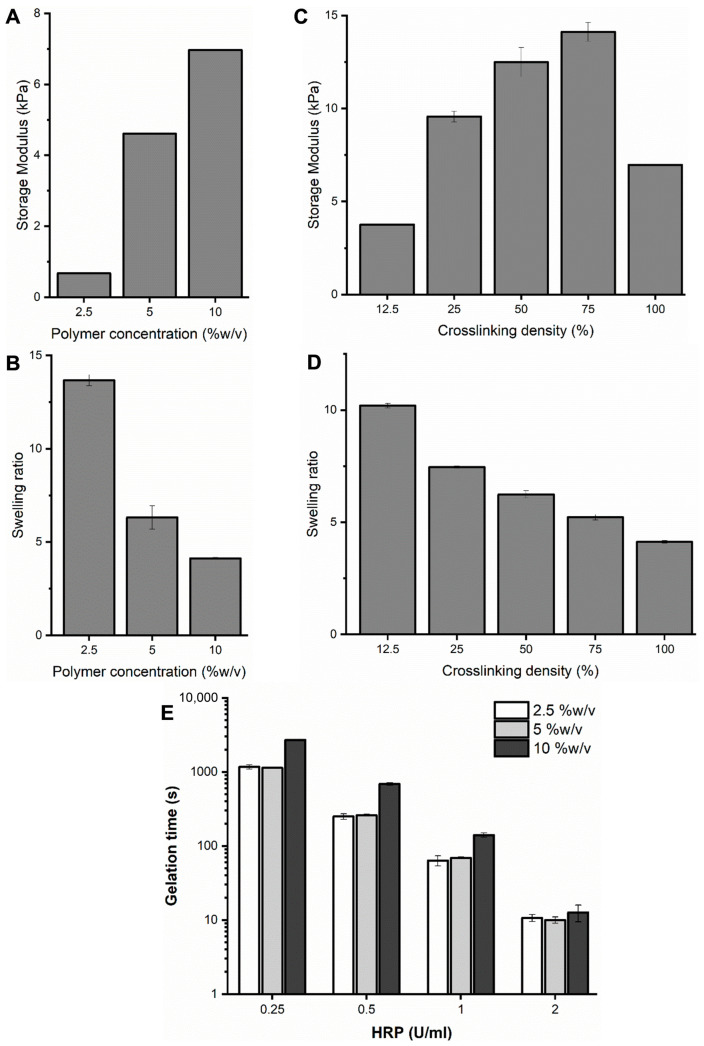
Characterization of DexTA–Mal hydrogels. (**A**) Storage modulus and (**B**) swelling ratio of hydrogels as a function of polymer concentration. Hydrogels were prepared using 4 U/mL of HRP and a H_2_O_2_/TA molar ratio of 0.5. (**C**) Storage modulus and (**D**) swelling ratio of 10% (*w*/*v*) hydrogels as a function of degree of crosslinking, and (**E**) gelation time based on HRP concentration [32].

DexTA–Mal hydrogels were prepared by incorporating HRP and H_2_O_2_ into the polymer solution within a mold. The swelling ratios of the resulting hydrogels were assessed after 24 h of immersion in PBS, revealing a predictable trend: higher polymer concentrations and increased crosslinking densities correlated with a lower swelling ratio (Figure 3B,D). Hydrogels were synthesized at a polymer concentration ranging from 2.5% to 10% (*w*/*v*) with varying degrees of crosslinking.

The degree of crosslinking profoundly impacts the mechanical properties of dextran-conjugated hydrogels. Advantageously, the stiffness of these hydrogels can be modulated by adjusting both the polymer concentration and the amount of H_2_O_2_ used in the crosslinking process (Figure 3A,C). This tunability allows the reproducible production of soft, medium, and stiff hydrogels by controlling the crosslinking density. The ability to fine-tune hydrogel stiffness is critical, as it facilitates controlled mechanotransduction between encapsulated cells and the surrounding hydrogel matrix.

Gelation time was determined using the vial tilting method. After systematically evaluating the hydrogel formation and properties based on the polymer concentration, HRP concentration, and crosslinking ratio (H_2_O_2_:TA ratio), as depicted in Figure 3, a polymer concentration of 10% *w*/*v* was selected for the subsequent experiments. At this concentration, the addition of 1.0 U/mL provided a suitable working time for further experimental procedures (Figure 3E).

**Figure 4:**
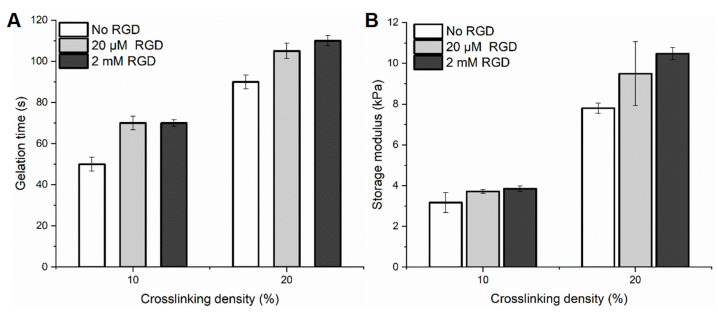
(**A**) Gelation time and (**B**) storage moduli of DexTA–Mal, depending on crosslinking density and RGD addition.

**Figure 5:**
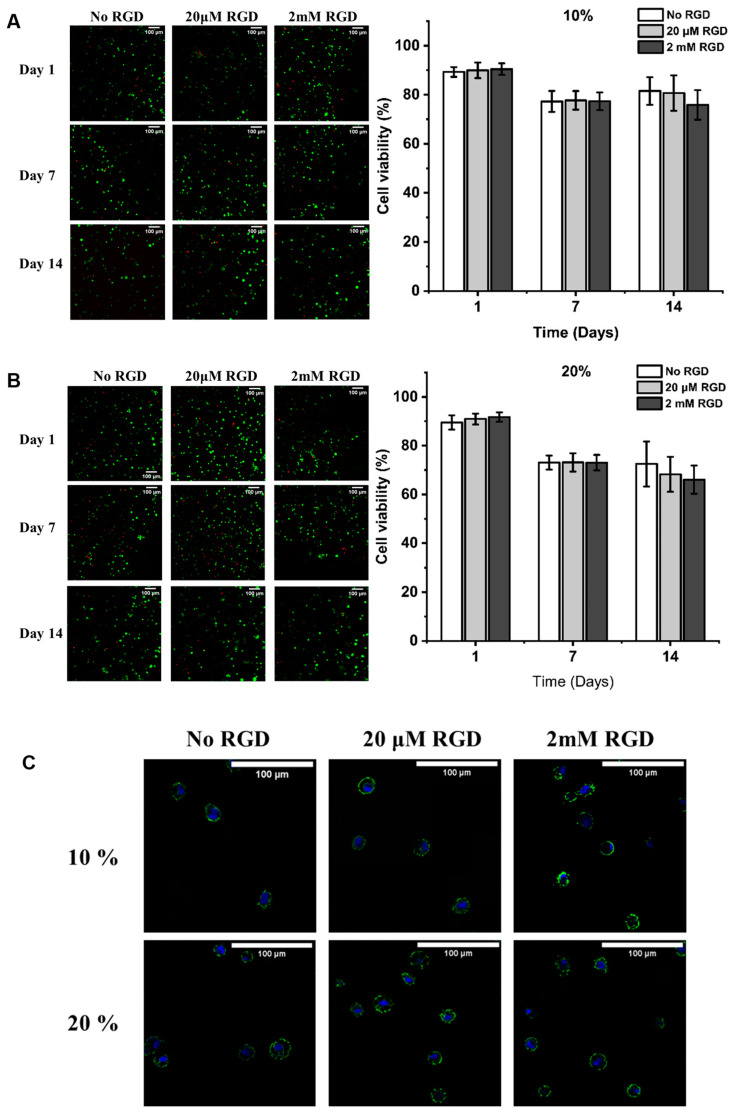
(**A**,**B**) Viability of hMSCs on days 1, 7, and 14, (Live (green) and dead (red) cells are stained).and (**C**) morphology of hMSCs on day 7 in injectable DexTA–Mal hydrogel compositions with 10% or 20% crosslinking, 1.0 U/mL HRP with or without RGD (Actin filaments (green) and nucleus (blue) are stained). Scale bar is 100 μm.

**Figure 6:**
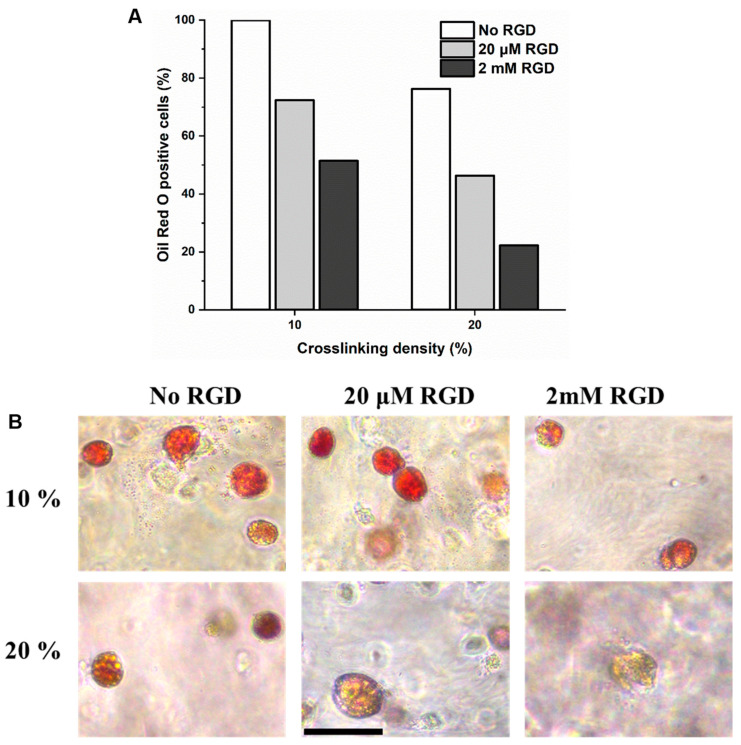
Adipogenic differentiation: (**A**) percentage of cells stained by Oil Red O in representative samples of DexTA–Mal hydrogels ± RGD, with 10 or 20% crosslinking density and hMSCs cultured for 14 days in bipotential medium. (**B**) Micrographs of representative samples of DexTA–Mal hydrogels ± RGD, with 10 or 20% crosslinking density and hMSCs cultured for 14 days in bipotential medium, stained with Oil Red O (red colour indicates fat deposition). Black scale bar: 100 inches.

In the subsequent experiments (Figure 4, Figure 5 and Figure 6), the concentration of H_2_O_2_ was adjusted to achieve crosslinking of 10% or 20% of the available tyramine groups. It was observed that the mechanical properties of the hydrogel, specifically its stiffness, could be finely tuned by varying the H_2_O_2_ concentration. However, at crosslinking levels exceeding 25%, the homogeneity of the gel decreased significantly. At higher degrees of crosslinking, the gel’s stiffness diminished due to the excessive H_2_O_2_, which interfered with the gelation process by inactivating the horseradish peroxidase (HRP) (Figure 3C) [34,35]. Notably, when crosslinking surpassed 75%, there was a marked reduction in the storage modulus which was attributed to the formation of intramolecular crosslinks. These intramolecular crosslinks reduce the number of effective elastic network chains, thereby diminishing the gel’s overall mechanical integrity [36].

Although low H_2_O_2_ concentrations are advantageous, especially in minimizing potential cytotoxic effects [16], it is essential to consider that rapid gelation is critical for in situ hydrogel formation. Slow gelation in vivo poses significant risks, including the diffusion of hydrogel precursors or bioactive molecules into the surrounding tissues and the potential failure of hydrogel formation.

### 2.4. Mechanical Properties of Selected Compositions

The mechanical properties of the DexTA–Mal hydrogels were evaluated under various conditions, including those used for cell-laden hydrogels (Figure 4). The storage modulus of DexTA–Mal hydrogels, a key indicator of the gel’s mechanical strength, was observed to increase with increasing crosslinking density. The introduction of the RGD peptide not only enhanced the storage modulus, but also affected the gelation time. Specifically, when a low concentration of RGD (20 μM) was added, the storage modulus increased from 3.2 to 3.7 kPa at a 10% crosslinking density, and from 7.8 to 9.5 kPa at a 20% crosslinking density. A further increase in RGD concentration, from 20 μM to 2 mM, led to a slight but notable increase in the storage modulus from 3.7 kPa to 3.8 kPa with 10% crosslinking, and from 9.5 kPa to 10.5 kPa with 20% crosslinking.

Overall, the DexTA–Mal hydrogels exhibited elastic properties, with two distinct storage modulus ranges: approximately 3.5 kPa for the softer gels (10% crosslinking) and 9.5 kPa for the stiffer gels (20% crosslinking). In addition to rheological assessments, compression testing was conducted, where the hydrogels were subjected to 30% strain (Figure S3). The results indicated that the Young’s modulus for the 10% crosslinked hydrogels was around 16 kPa, with minimal influence from RGD addition. In contrast, for the 20% crosslinked hydrogels, the Young’s modulus ranged from 62 kPa to 83 kPa, increasing with the addition of RGD and further reinforcing the stiffness of the hydrogel network.

### 2.5. Viability and Morphology of hMSCs in the Different Hydrogels

The viability of human mesenchymal stem cells (hMSCs) encapsulated within DexTA–Mal hydrogels was assessed via a live/dead assay using calcein and ethidium homodimer staining (Figure 5A–C). The resulting micrographs demonstrated a uniform distribution of hMSCs throughout the DexTA–Mal hydrogels.

On Day 1, over 88% of hMSCs remained viable in the DexTA–Mal hydrogels without RGD. When 20 μM or 2 mM RGD was incorporated, the cell viability in the hydrogels with comparable crosslinking densities showed a slight increase compared to those without RGD. After 7 days, the cell viability in the hydrogels with 10% and 20% crosslinking densities decreased to 77% and 73%, respectively, regardless of the RGD concentration. Across all conditions, the hMSCs retained a rounded morphology, unaffected by the presence or absence of RGD (Figure 5C).

Cell viability remained consistent over a 14-day period, with a minimal impact from RGD addition. Interestingly, the highest viability was observed in the absence of RGD. These cell-laden DexTA–Mal hydrogels support a biocompatible environment conducive to cell survival, proliferation, differentiation [37], and matrix formation.

### 2.6. Evaluation of In Vitro Adipogenesis of hMSCs Encapsulated in DexTA–Mal Hydrogels

To evaluate the adipogenic and osteogenic differentiation, MSC-laden DexTA–Mal hydrogels were cultured for 14 days in a bipotential medium. Adipogenic differentiation was confirmed by positive Oil Red O staining, which revealed significant fat deposition within the hydrogels. In contrast, osteogenic differentiation was not evident, as indicated by the lack of clear Alizarin Red staining.

Oil Red O staining further demonstrated that MSCs encapsulated in the softer DexTA–Mal hydrogels (10% crosslinking) without RGD groups exhibited the highest accumulation of lipid droplets (Figure 6). This observation supports the hypothesis that a softer microenvironment promotes adipogenesis more effectively than a stiffer one [38]. Additionally, the average number of lipid droplets decreased with an increasing RGD concentration. This phenomenon can be attributed to the absence of receptors for dextran on the cells, limiting their ability to sense the hydrogel’s mechanical properties. The incorporation of RGD peptides provides the material with binding sites for integrin receptors, thereby enhancing the cells’ ability to sense and respond to the mechanical properties of the hydrogel.

These findings are consistent with previous studies, which have demonstrated that DexTA-based microenvironments favour adipogenic differentiation [38,39]. However, no clear osteogenic differentiation was observed with Alizarin Red staining, which may be attributed to the relatively low hydrogel stiffness. Osteogenic differentiation of MSCs requires a specific mechanical microenvironment with appropriate stiffness ranges, preferably 11–30 kPa in alginate or poly(ethylene glycol) (PEG) hydrogels [40]. Similar outcomes were observed in Dex–TA cell-laden microgels [41].

## 3. Conclusions

The synthesis of dextran–tyramine–maleimide (DexTA–Mal) was successfully achieved, demonstrating its effectiveness in hydrogel formation through tyramine-based enzymatic crosslinking. This bifunctional dextran proved to be highly versatile, allowing functionalization with biomolecules such as the CRGDS peptide and enabling the production of hydrogels with tuneable mechanical properties simply by adjusting the crosslinking density via H_2_O_2_ concentration. Such tunability makes these hydrogels highly suitable as 3D polymer scaffolds for cell culture and differentiation applications. Additionally, this hydrogel platform shows strong potential for delivering bioactive peptides and proteins, particularly for immobilizing biomolecules like cell adhesion ligands and growth factors in tissue engineering.

Encapsulation of human mesenchymal stem cells (hMSCs) within DexTA–Mal hydrogels via enzymatic crosslinking preserved high cell viability throughout a 14-day in vitro culture period. The ability of these hydrogels to support successful adipogenic differentiation further underscores their potential as scaffolds for soft tissue engineering.

Future studies should investigate stiffer DexTA–Mal hydrogels for osteogenic differentiation to further explore their potential. Overall, this hydrogel system represents a promising and adaptable platform for applications in tissue engineering and regenerative medicine.

## 4. Materials and Methods

### 4.1. Materials

Dextran 40 (40 kDa, pharmaceutical grade) was purchased from Pharmacosmos, Denmark. Dithiol PEG (3.4 kDa) was purchased from Creative PEGWorks, Durham, NC, USA. Tyramine (99.0%), N,N-Dimethylformamide (DMF, anhydrous, 99.8%), LiCl (99.0%), p-nitrophenyl chloroformate (96.0%), *n*-Boc-1,4-diaminobutane (≥97.0%), pyridine (anhydrous, 99.8%), DMSO-d_6_ (99.9%), sodium hydroxide (NaOH, ≥97.0%), tris(2-carboxyethyl)phosphine hydrochloride (TECP, ≥98.0%), trifluoroacetic acid (TFA, ≥99.0%), bovine serum albumin (BSA, ≥98.0%), horseradish peroxidase (HRP, 325 units/mg solid), ascorbic acid 2-phosphate (ASAP), insulin, IBMX, indomethacin, and hydrogen peroxide (30%) were purchased from Sigma-Aldrich (St. Louis, MO, USA). N-(α-maleimidoacetoxy)succinimide ester and 4-(4,6-Dimethoxy-1,3,5-triazin-2-yl)-4-methylmorpholinium chloride (DMTMM, 97.0%) were purchased from Fluorochem Ltd., Hadfield, UK. Coomassie brilliant blue R 250, ethyl acetate (EtOAc, ≥99.5%), n-Hexane (≥95.0%), ethanol (≥99.9%), and diethyl ether (≥99.7%) were purchased from Merck, Kenilworth, NJ, USA. FITC-labelled peptide was purchased from Pepscan, Lelystad, The Netherlands. Penicillin and streptomycin were purchased from Invitrogen, Carlsbad, CA, USA. α-MEM, DMEM, FBS, and glutaMAX were obtained from Gibco, Billings, MT, USA. Milli-Q water was used from the Milli-Q Advantage A10 system, equipped with an 0.22 μm Millipak^®^-40 Express filter (Merck, Kenilworth, NJ, USA). CRGDS peptide was obtained from GenScript, Rijswijk, The Netherlands.

### 4.2. Synthesis of Dextran-Tyramine-Maleimide

- Dextran-p-nitrophenyl carbonate (Dex–PNC)

Dextran was reacted with p-nitrophenyl chloroformate (PNC) to form p-nitrophenyl carbonate conjugates, which were then treated with primary amine-containing compounds [16]. LiCl (4.0 g, dried at 115 °C) and dextran (5.0 g, 30.8 mmol r.u.) were weighed into a 500 mL three-necked round-bottom flask equipped with a stirrer bar. The flask was evacuated and refilled with nitrogen 3 times, after which it was left under vacuum conditions at 95 °C for 1.5 h. After thoroughly drying, the flask was filled with nitrogen and 200 mL of anhydrous DMF was added via a cannula while stirring. The flask was then equipped with a thermometer and heated to 95 °C while stirring the solution. Once the dextran was completely dissolved, the solution was cooled to 0 °C and anhydrous pyridine (2.0 mL, 25.8 mmol) was added. Subsequently, freshly sublimed PNC (2.50 g, 12.4 mmol) was added in small portions, keeping the temperature below 2 °C. After 1 h, the reaction mixture was poured into 1.0 L of ice-cold ethanol. The precipitate was filtered off (Por 4) and washed with copious amounts of cold ethanol and diethyl ether. After drying under vacuum conditions, the product was obtained as a white powder (6.00 g, 92.6% yield, DS 29). As shown in Figure S1, ^1^H-NMR (400 MHz, DMSO-d_6_): δ(ppm) = 3.0–4.0 (saccharide ring protons, m, 6H), 4.2–5.8 (anomeric and hydroxyl protons, m, 4H), 7.58 (Ar o-CH, d, 2H), and 8.34 (Ar m-CH, d, 2H).

b. Dextran–tyramine–butylamine (DexTA–bNH_2_)

DexTA–bNH_2_ was synthesized as previously reported [2]. Typically, Dex–PNC_29_ (5.00 g, 23.8 mmol r.u, 6.90 mmol PNC) was weighed into a 250 mL three-necked round-bottom flask equipped with a stirrer bar. The flask was evacuated and refilled with nitrogen for 3 times, after which 100 mL of anhydrous DMF was added via a cannula while stirring. Once the Dex–PNC was completely dissolved, *n*-Boc-1,4-diaminobutane (0.326 g, 1.73 mmol) was added under a nitrogen flow and the reaction was allowed to proceed for 20 min, then tyramine (0.473 g, 3.45 mmol) was added. After 1 h, the reaction mixture was poured into 1.0 L of ice-cold ethanol. The precipitate was filtered off (Por 4) and washed with copious amounts of cold ethanol and diethyl ether. After drying under vacuum conditions, the crude product was obtained as a white powder (4.38 g). As shown in Figure S2, ^1^H-NMR (400 MHz, DMSO-d_6_): δ(ppm) = 1.2–1.5 (methyl protons and -CH_2_-**(*CH*_2_)_2_**-CH_2_-protons, m, 13H), 3.0–4.0 (saccharide ring protons, m, 6H), 4.2–5.4 (anomeric and hydroxyl protons, m, 4H), 6.67 (Ar m-CH, d, 2H), and 6.99 (Ar o-CH, d, 2H).

In the second step, DexTA–(bNHBoc) (4.58 g) was dissolved in 50 mL of Milli-Q water. Trifluoroacetic acid (TFA) (5.0 mL) was added, then the mixture was stirred overnight.

The reaction was neutralized by 1.0 M NaOH solution and dialysed against Milli-Q water for 4 days (MWCO 3500 Da), followed by filter sterilization and freeze-drying to yield the product (DexTA–bNH_2_) as a white foam (3.85 g, DS (TA) 9.0%, DS (-NH_2_) 5.2%, 91.6% yield). ^1^H-NMR (400 MHz, DMSO-d_6_): δ(ppm) = 1.4–1.6 (-CH_2_-**(*CH*_2_)_2_**-CH_2_-protons, m, 4H), 3.0–4.0 (saccharide ring protons, m, 6H), 4.2–5.4 (anomeric and hydroxyl protons, m, 4H), 6.67 (Ar m-CH, d, 2H), and 6.99 (Ar o-CH, d, 2H).

c. Dextran–tyramine–(butylamine)maleimide (DexTA–Mal)

DexTA–bNH_2_ (3.80 g, 1.12 mmol -NH_2_) was weighed into a 250 mL three-necked round-bottom flask equipped with a stirrer bar. The flask was filled with nitrogen and 100 mL of anhydrous DMF was added via a cannula while stirring. Once the DexTA–bNH_2_ was completely dissolved, Et_3_N (0.388 g, 3.84 mmol) and *n*-(α-maleimidoacetoxy)succinimide ester (AMAS, 0.645 g, 2.56 mmol) were added. The mixture was stirred for 24 h at room temperature under a nitrogen atmosphere. After 24 h, the reaction mixture was poured into 1.0 L of ice-cold ethanol. The precipitate was filtered off (Por 4) and washed with copious amounts of cold ethanol and diethyl ether. After drying under vacuum conditions, the crude product was obtained as a white powder. The crude product was then dissolved in water and dialysed against Milli-Q water for 3 days (MWCO 3500 Da), followed by filter sterilization and freeze-drying to yield the product as a white foam (3.72 g, DS (Mal) 5%, 94.3% yield). ^1^H-NMR (400 MHz, DMSO-d_6_): δ(ppm) = 3.0–4.0 (saccharide ring protons, m, 6H), 4.2–5.4 (anomeric and hydroxyl protons, m, 4H), 6.67 (Ar m-CH, d, 2H), 6.99 (Ar o-CH, d, 2H), and 7.08 (maleimide protons, d, 2H) [32].

The calculation of the DS of -PNC, -TA, -bNH_2_, and -Mal is based on the integrals of δ 4.2–5.8 ppm (corresponding to the 4 anomeric and hydroxyl protons from dextran), compared with the integral of the aromatic 4 protons of p-nitrophenyl (7.40–7.65 ppm and 8.20–8.40 ppm) or tyramine (6.60–6.75 and 6.90–7.07 ppm), the 4 protons of (-CH_2_-**(*CH*_2_)_2_**-CH_2_NH_2_ (1.40–1.60 ppm), and the 2 protons of maleimide (6.91–7.04) for Dex–Mal, respectively. The DS of dextran is given as the percentage of saccharide units modified in dextran.

### 4.3. Hydrogel (Bio-)Functionalization

The reactivity of the maleimide moieties functionalized to dextran was proven through hydrogel formation via thiol–maleimide Michael-type addition. Michael-type addition dextran hydrogels of DexTA–Mal at a total polymer concentration of 10% *w*/*v* were formed by reacting maleimide functionalized dextrans with poly(ethylene glycol) dithiol (MW = 3400, Creative PEGWorks, Winston Salem, NC, USA) at a 1:1 maleimide: thiol molar ratio in PBS (pH = 7.4). Biofunctionalization was tested by conjugation of a FITC-labelled peptide or BSA to DexTA-b(NH)Mal. The FITC-labelled peptide and BSA were reduced in PBS by the addition of a 2.5-fold molar excess of TCEP and incubation for 1 h at 37 °C. Afterwards, the reduced FITC-labelled peptide or BSA was added to a DexTA–b(NH)Mal solution in PBS in a 1:10 molar ratio to produce Dex–peptide and Dex–BSA conjugates, respectively. The reaction mixtures were gently shaken for one hour at room temperature. The unreacted maleimide moieties were blocked by th addition of excess cysteine. The unreacted FITC-labelled peptide and the successful Dex–peptide conjugate were then separated on an SDS-PAGE gel under reducing conditions and imaged by the FluorChem M system (ProteinSimple). To analyse the Dex–BSA conjugate, protein bands were visualized by Coomassie blue staining after the separation of the BSA and Dex–BSA conjugate by SDS-PAGE.

### 4.4. Hydrogel Formation and Gelation Time

Enzymatically crosslinked hydrogels of the tyramine-conjugated polymers (DexTA, DexT–Mal, and DexTA–Mal) at a total polymer concentration of 2.5% *w*/*v*, 5% *w*/*v*, and 10% *w*/*v* were prepared in vials at room temperature. In a typical procedure, after dissolving the tyramine-conjugated polymers in PBS, various concentrations of HRP (HRP/TA ratio, series ranging from 0.0625 to 0.5 mg/mmol) were added to the polymer solutions and incubated overnight at 4 °C. Subsequently, a freshly prepared PBS solution of H_2_O_2_ was added to the HRP-containing polymer solution and immediately mixed using a vortex mixer. The time to form a gel was determined using the vial tilting method. The experiment was performed in triplicate.

### 4.5. Hydrogel Swelling

Three hydrogels per condition were formed in an 8 mm diameter × 1.5 mm height Polytetrafluoroethylene (PTFE) mold. After crosslinking, hydrogels were allowed to freely swell in PBS for 24 h in order to reach the swelling equilibrium. Swollen hydrogels mass ($w_{s}$) were weighted after the removal of surface water.

The polymer content ($w_{p}$) in the hydrogel was calculated using the following equation:

$$w_{p}=\pi r^{2}h\times weight\;per\;volume\;polymer$$

(r: radius of PTFE mold; h: height of PTFE mold)

The swelling ratio was calculated as follows: $\frac{w_{s}-w_{p}}{w_{p}}$.

### 4.6. Mechanical Properties

The viscoelastic properties of these cylindrical DexTA–Mal hydrogels were measured on the HR 20 Discovery Hybrid Rheometer-TA instruments (New Castle, DE, USA), equipped with a Peltier stage temperature control system and parallel plate (PP ø 8 mm) geometry. Measurements were taken at 20 °C (±0.2 °C) under an initial normal force of 0.05 N, within the linear viscoelastic (LVE) range, at 0.5% strain and 1.0 Hz frequency. The hydrogel samples were equilibrated for 24 h in 1 mL PBS at 4 °C before the measurement. A minimum of three hydrogel samples were measured for each condition.

### 4.7. Compression Test

Compression testing was performed on the cylindrical DexTA–Mal with or without RGD peptide hydrogel samples (described in “Hydrogel formation”) with the use of the HR 20 Discovery Hybrid Rheometer-TA instruments (USA). The hydrogels underwent a single compression cycle with a maximum strain of 30% using a compression speed of 0.05 mm/s. The compression tests were conducted at room temperature, and at least three specimens were tested for each formulation.

### 4.8. Cell Culture and Expansion

The mesenchymal stem cells were isolated from human tissues obtained during total knee replacement surgeries. The patients gave informed consent to use these cells for research and the steps for cell isolation and proper usage were reviewed and approved by the ethics committee. These human bone marrow-derived MSCs were isolated as previously described [42] and cultured in an MSC adipogenic and osteogenic differentiation (bipotential) medium (α-MEM and DMEM, supplemented with 10% FBS, 1% glutaMAX, 0.1 mM ascorbic acid 2-phosphate (ASAP), 100 U/mL penicillin and 100 µg/mL, 5 µg/mL insulin, 0.25 mM IBMX, 0.1 mM indomethacin, and 100 nM dexamethasone (added fresh). The use of human material was approved by a local medical ethical committee. The medium was refreshed twice a week, and 10 million cells per 1 mL were used for the experiments at 80% confluency and in passage 3.

- Cell encapsulation in DexTA–Mal Hydrogels

DexTA–Mal was dissolved in PBS and incubated with HRP overnight at 4 °C. Then, the DexTA–Mal solution with HRP was divided into three equal volumes to prepare the no RGD, 20 µM RGD, or 20 mM RGD conditions. Each vial was further divided into 2 groups for crosslink densities. For cell-laden hydrogel preparation, hMSCs were added to yield a concentration of 10 million cells/mL in the hydrogel precursors. Freshly prepared H_2_O_2_ for 2 different crosslinking ratios (10 and 20%) were added to the mixtures (with or without cells), vortexed, and pipetted directly into a PTFE mold to form identical cylindrical hydrogels (8 mm diameter and 1.5 mm height; the final concentration in the hydrogels was 10% *w*/*v* DexTA–Mal, 1.0 U/mL HRP and H_2_O_2_ for 10 or 20% crosslinking).

b. Live/Dead Cell Viability Assay

The cell-laden hydrogels were evaluated with the live/dead cell viability assay (Invitrogen) according to the manufacturer’s instructions on days 1, 7, and 14.

All samples were incubated at 37 °C and 5% CO_2_ for 30 min in the live/dead solution (Alexa Fluor-488 Calcein AM and Alexa Fluor-568 Ethidium homodimer) and observed with fluorescent confocal microscopy (Laser Scanning Microscope 880, Zeiss, Oberkochen, Germany). The objective EC Plan-Neofluar 10×/NA 0.3 was used for this study. The single images were randomly selected from different areas of each sample.

Digital images were processed using the FIJI 2.14.0 image processing software. Finally, an index of live cells was calculated from the ratio of the live cell (green) number to the total cell number (green + red) from each area. Values represent the mean ± standard deviation of at least 3 biological replicates.

c. Influence of Hydrogel Formulations on Morphology in the DexTA–Mal Hydrogels

Assessment of the cell shape of hMSCs after the formation of DexTA–Mal hydrogels with or without RGD peptide was carried out. The hydrogels were formed as illustrated above with the same concentration of the polymers (10% *w*/*v* DexTA–Mal). A single hydrogel per well was added to a 12-well plate in triplicate with a cell concentration of 10 million cells/mL. Morphology was assessed on day 7 as the cultured cell-laden DexTA–Mal hydrogels were fixed with 4% *w*/*v* paraformaldehyde, permeabilized with 0.5% v/v Triton X 100, and stained with 0.25% Alexa Fluor- 488 Phalloidin (F-actin), as well as 1% Alexa Fluor-405 DAPI. The same confocal laser scanning microscope with objective C-Apochromat 40×/1.2 W Korr FCS M27 was used for this study. The single images were randomly selected from different areas of each sample and these images were processed using FIJI software. The cell shape was captured by the presence of the actin filament (green) and nucleus (blue) from each area. These images were processed using FIJI software.

d. Differentiation

Cell-laden DexTA–Mal hydrogels were washed with PBS and fixed with 10% buffered formalin. Adipogenic differentiation was analysed by staining the samples with a freshly prepared and filtered Oil Red O, then visualized using brightfield microscopy. Osteogenic differentiation was analysed by staining samples with filtered Alizarin Red in distilled water, then visualized using brightfield microscopy.

## Data Availability

The data presented in this study are available on request from the corresponding author.

## Supplementary Materials

The following supporting information can be downloaded at: https://www.mdpi.com/article/10.3390/gels10090566/s1, Figure S1: Synthesis route (A) and ^1^H NMR spectra in DMSO-d_6_ (B) of Dex–PNC; Figure S2: ^1^H NMR spectra of DexTA–bNHboc (B) in DMSO-d_6_; Figure S3: Stress and strain curves of the cylindrical DexTA–Mal hydrogel samples with a crosslinking density of (a) 10% and (b) 20%.

## Abbreviations

3D	Three-dimensional
AMAS	n-(α-maleimidoacetoxy) succinimide ester
BSA	Bovine serum albumin
Cys	Cysteine
EC	Enzymatic crosslinking
Dex	Dextran
DexTA	Dextran–tyramine
DexTA–Mal	Dextran–tyramine–maleimide
DMF	N,N-Dimethylformamide
DMSO	Dimethyl sulfoxide
DS	Degree of substitution
ECM	Extracellular matrix
hMSCs	Human mesenchymal stem cells
HRP	Horseradish peroxidase
H_2_O_2_	Hydrogen peroxide
^1^H-NMR	Proton nuclear magnetic resonance
LVE	Linear viscoelastic range
MW	Molecular weight
MWCO	Molecular weight cut-off
PBS	Phosphate-buffered saline
PEG	Polyethylene glycol
PNC	p-nitrophenyl chloroformate
PTFE	Polytetrafluoroethylene
RGD	Arginylglycylaspartic acid
TA	Tyramine
