# Supplementary material for: Bioactive Hydrogels Based on Tyramine and Maleimide Functionalized Dextran for Tissue Engineering Applications
Source: Gels. 2024 Aug 30;10(9):566. doi: 10.3390/gels10090566 (PMC11431488; doi:10.3390/gels10090566)
Supplement: Supplementary file 1 [file gels-10-00566-s001.zip › gels-3113410-supplementary.pdf]

Supplementary information

A

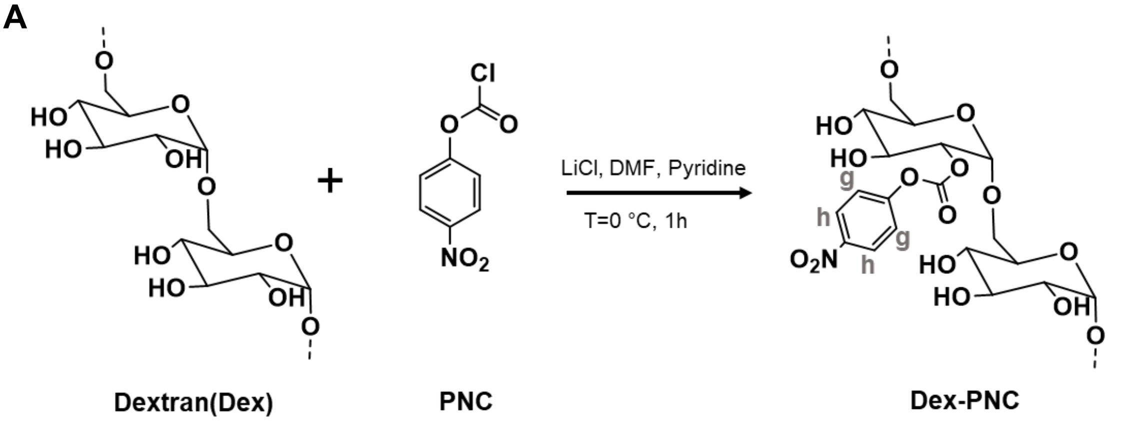

B

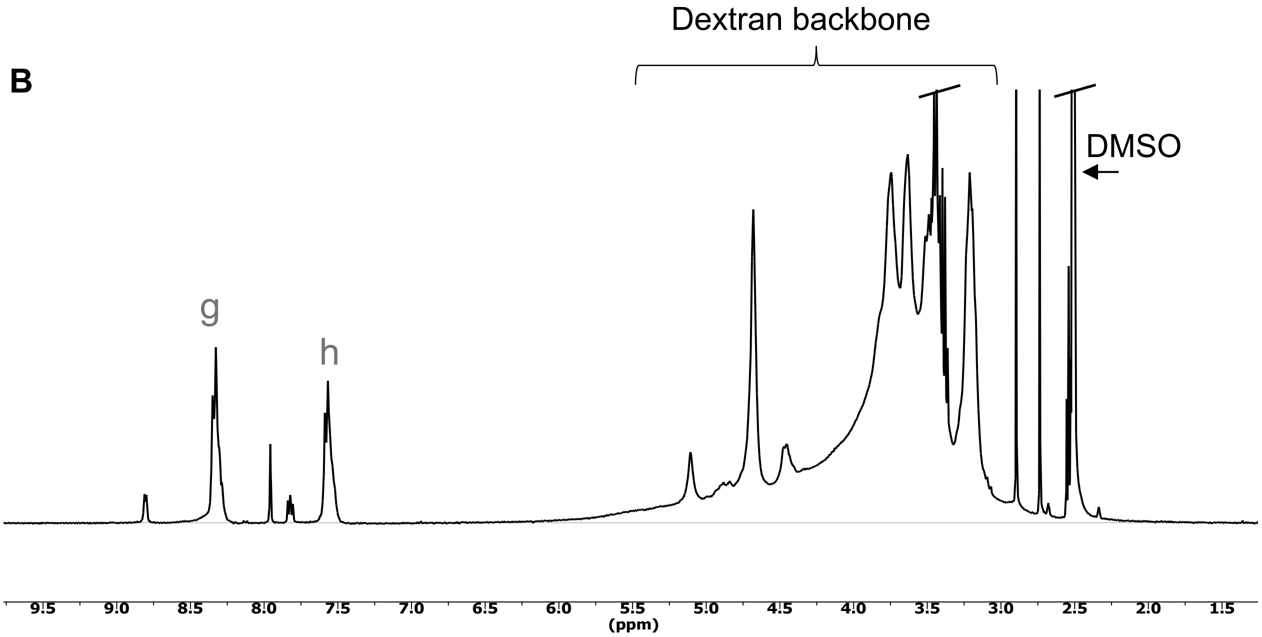

Figure S1: Synthesis route (A) and ^{1}H NMR spectra in DMSO-d_6 (B) of Dex-PNC.

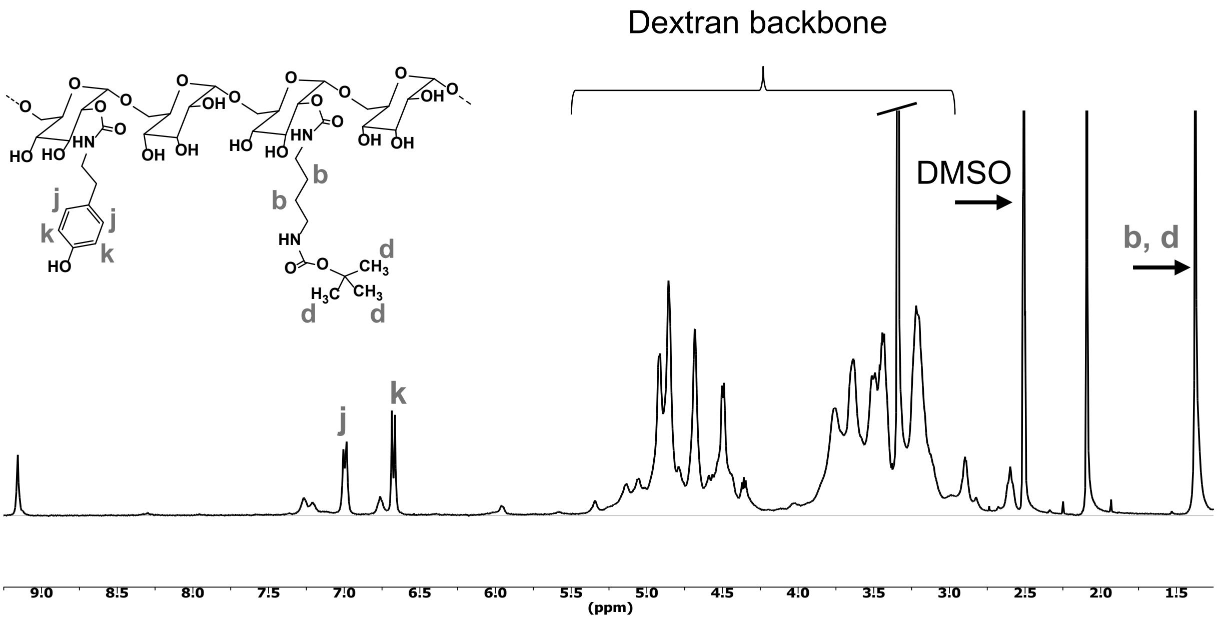

Figure S2: ^{1}H NMR spectra of Dex-TA-bNHboc(B) in DMSO-d_6.

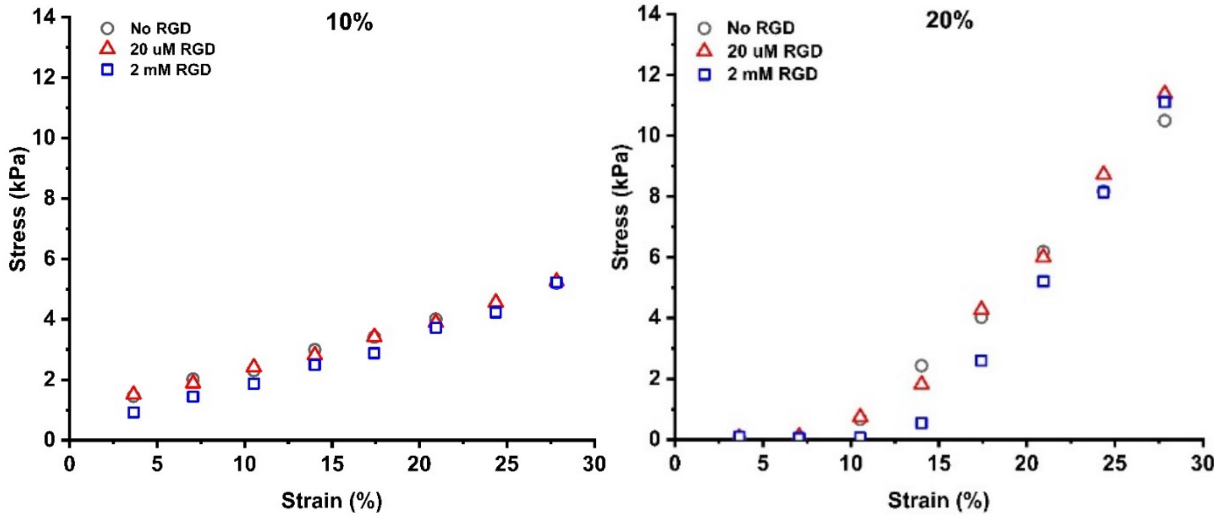

Figure S3: Stress and strain curves of the cylindrical Dex-TA-(bNH)Mal hydrogel samples with a crosslinking density of a) 10% b) 20%.
